# Combination Therapies in Ophthalmology: Implications for Intravitreal Delivery

**Published:** 2011-01

**Authors:** Gholam A. Peyman, Kamran Hosseini

**Affiliations:** 1 University of Arizona, Phoenix, Arizona, USA; 2 Tulane University, New Orleans, Louisiana, USA; 3 InSite Vision Inc., Alameda, California, USA

**Keywords:** Combination Therapy, Combined Modality Therapy, Intravitreal Delivery

## Abstract

Most pathological processes involve complex molecular pathways that can only be modified or blocked by a combination of medications. Combination therapy has become a common practice in medicine. In ophthalmology, this approach has been used effectively to treat bacterial, fungal, proliferative/neoplastic, and inflammatory eye diseases and vascular proliferation. Combination therapy also encompasses the synergistic effect of electromagnetic radiation and medications. However, combination therapy can augment inherent complications of individual interventions, therefore vigilance is required. Complications of combination therapy include potential incompatibility among compounds and tissue toxicity. Understanding these effects will assist the ophthalmologist in his decision to maximize the benefits of combination therapy while avoiding an unfavorable outcome.

## INTRODUCTION

Combining means and therapeutics to address various pathologic processes is now a familiar topic across many, if not all, medical specialties. There is compelling clinical, technological and commercial justification for combining therapies into fixed and/or unfixed drug combinations or combining a device and a drug to achieve better outcomes. Usually, fixed drug combinations are formulated to address patient compliance and convenience or manufacturing costs. Other fixed therapies encompass the combination and engineering of drug and device into one unit as a means of drug delivery or combining a drug and a physical source of energy (thermal or certain wavelengths) to achieve a localized therapeutic effect. Examples are cardiovascular drug eluting stents^1^, intravitreal drug implants^2^, diabetes products, iontophoretic transdermal patches^3^, and photodynamic therapy. Such drug and device combinations are mainly aimed at realizing considerable therapeutic advantages as compared to administering a single drug, or the drug and the device separately.

There are also convincing medical and biological reasons to combine two or more drugs. Different pathologies develop and progress through multiple molecular pathways and therefore, different mechanisms of action and opportunities exist to interfere and partially block such pathways. Capitalizing on such opportunities must be done with extreme care, since combining chemical entities and formulations can be toxic to specific cells, and sometimes to whole organs. Although such combinations may result in additive or synergistic therapeutic actions, they may also cause unfavorable outcomes; therefore stringent pharmacovigilance at all times is warranted.

Awareness of the potential hazards of combination products has prompted many regulatory bodies such as the US Food and Drug Administration (FDA), European Medicines Agency (EMA), and the Japan Health Agency to mandate thorough screening of potential benefits and adverse effects. These measures have resulted in new guidelines and policies for fixed therapeutic products.^4^ However, there is considerably less control and vigilance present/ possible at the level of the practicing clinician for unfixed combinations (administering two or more drugs at variable doses and frequencies). There are hazards, and therefore physicians need to be aware of the potential risks and continue to share their findings with the medical community.

Discussing the broad ramifications of all combination products would go beyond the scope of this paper. Therefore, we focus on combination drugs in ophthalmology and in particular, on intravitreal drug combinations.

The eye is a unique structure, in which different tissues (nerve, muscle, connective tissue, aqueous and vitreous humor, etc.) interact and fuse in a compact space with well-defined boundaries. The eye is susceptible to pathologies that can affect individual tissues. Pathological processes may also affect more than one tissue at a time and therefore, multiple drugs could be used to address each layer and tissue accordingly. Our current knowledge of many ophthalmic diseases is still incomplete regarding the exact causative molecular pathways. Current clinical practice categorizes a collection of disorders under single disease entities, e.g., glaucoma, dry eye, age-related macular degeneration. However, in the abovementioned pathologies, varying stages and severities of disease manifestations are the result of a collection of molecular pathways brought about by multi-factorial conditions. As our basic knowledge of these conditions grows, so do our therapeutic strategies to address them. Precise mapping of different molecular pathways allows us to devise approaches for each independent pathway. It is no surprise that no single drug can address all pathways. Therefore, combination approaches have become inevitable, and understanding the chemical and physical interactions of the components remains of paramount importance. Disregarding such considerations may result in irreversible consequences.

Combination of drug and device within ophthalmology dates back to the 1970s when the first drug and device combination was approved in the USA. Ocusert (Alza Corporation, Palo Alto, CA, USA) was a pilocarpine-containing, polymer-membrane unit that provided a depot in the conjunctival cul-de-sac to address glaucoma.^5^ Since then, other ocular drug/ device implants such as Vitrasert and Retisert (Bausch & Lomb Inc., Rochester, NY, USA), both intravitreal devices, have been embraced in clinical practice. There is even an encapsulated cell delivery device (Neurotech USA Inc., Lincoln, RI, USA) in clinical development as an intravitreal implant which produces ciliary neurotrophic factor (CNTF), which can potentially provide benefit for patients with different retinal degenerative disorders.^6^ This device consists of living cells encapsulated within semi-permeable polymer membranes and supportive matrices. Recently, a new product was approved in the US that involves a drug and device combination (Ozurdex; Allergan Inc., Irvine, CA, USA) where the device injects a biodegradable dexamethasone depot intravitreally using a specialized disposable syringe and needle system.^7^

Peyman and colleagues^8^ have recently invented a drug and device combination where the device can inject a depot drug using jet propulsion force without needle entrance into the vitreous cavity, therefore, drastically reducing the chance of endophthalmitis.

There are other drug and device combinations in late-stage clinical development. Examples include contact lenses with drug coatings as drug delivery means for seasonal allergies and other anterior segment pathologies^9^,^10^, intraocular lenses with drug coatings as prophylaxis for post-cataract surgery, and drug coated punctal plugs to address compliance issues in glaucoma patients.^11^ Furthermore, there have been experiments with biodegradable punctal plugs to investigate antibiotic and steroid delivery potentials.^12^ Another form of drug device combination in ophthalmology is photodynamic therapy, where intravenous circulating drugs combined with focused irradiation of a certain wavelength of light results in activation of free radicals from that drug, enhancing a local coagulating effect on intruding choroidal/retinal vessels.^13^

## COMBINATIONS OF TWO OR MORE DRUGS

The combination of multiple chemical agents for ocular disorders provides an effective strategy to address clinical needs in acute and chronic conditions. When two or more different drugs are mixed *in vitro* or *in vivo*, they may interact. Some drugs change the way others are transported or absorbed in the tissue. Such interactions may occur during chemical and physical intraocular mixing or at the time of mixture of injections.

Drug interactions may increase or decrease drug effects. Competitive effects occur during combination of drugs which can be positive or negative, depending on whether the drugs have synergistic or antagonistic effects. Drug displacement interactions are a possibility; this can occur at binding or receptor sites. Alternatively, the combination of drugs can produce a new effect that neither drug produces on its own. The effect of combining drugs within an organ such as the eye can be considered localized. However, one needs to be alert towards the possible additional interactions with systemically dosed agents and thus, adverse interactions elsewhere in the body.

Ophthalmology is a specialty that is quite familiar with combinations of different drugs and therapies. As an example, endophthalmitis is a well known (and dreaded) condition that is currently addressed with unfixed combination therapy; at least two different classes of antibiotics are co-administered to provide coverage for both gram-negative and gram-positive bacteria.^14^ Another example includes artificial tears and cyclosporine formulations (Restasis; Allergan Inc., Irvine, CA, USA), which are increasingly being combined to treat a plethora of dry eye cases. Also, different classes of glaucoma drugs are being prescribed either in fixed combinations or unfixed and co-prescribed combinations by physicians to control intraocular pressure in glaucoma patients. Examples of fixed combinations for glaucoma include brinzolamide 1% plus timolol 0.5% ophthalmic suspension (Azarga; Alcon Laboratories Inc., Fort Worth, TX, USA), travoprost 0.004% plus timolol 0.5% (DuoTrav; Alcon Laboratories Inc., Fort Worth, TX, USA), a fixed combination of 0.005% latanoprost and 0.5% timolol (Xalacom; Pharmacia, Kalamazoo, MI, and Pfizer, New York, NY, USA), and a fixed combination of bimatoprost 0.03% plus timolol 0.5% (Ganfort, Allergan, Irvine, CA, USA). There are also a number of fixed ophthalmic combinations of corticosteroids and antibiotics. Well known examples include Tobradex (dexamethasone 1 mg/ml plus tobramycin 3 mg/ml; Alcon Laboratories Inc., Fort Worth, TX, USA), Zylet (loteprednol and tobramycin; Bausch & Lomb Inc., Rochester, NY, USA), and dexamethasone-netilmicin (dexamethasone 1 mg/ml plus netilmicin 3 mg/ml). There is also a fixed combination of dexamethasone 0.1% and a macrolide (azithromycin 1%) in Durasite (a polycarbophil-based ophthalmic delivery system) in late stage clinical development (ISV-502, InSite Vision, Alameda, CA, USA).

Next, we will briefly discuss possible physical-chemical interactions between various drugs and comment on their possible applications and/or outcomes for intravitreal combination therapies.

It remains almost impossible to predict drug interactions in different locations of an organ or the whole body, as transport and elimination routes and dynamics can vary greatly depending on ongoing physiological and pathological processes. However, there are a few exceptions. One of them is the vitreous body, where locally administered drugs can achieve high concentrations, at least for a certain period of time. It should be noted that the duration of this effect depends on the drug’s half-life, molecular weight, molecular size, electric charge, elimination route, etc.

Peyman and colleagues 15 used the combination of steroids and antibiotics in the vitreous body for the first time in 1974 to treat bacterial endophthalmitis. This combination approach followed their findings regarding diffusion processes governing the transport of peroxidase as a trace material in the posterior segment. They discovered that junctional complexes of the retinal pigment epithelium act as the limiting barrier of transport in both directions.^16^ This prompted them to conclude that intravitreal injections could be feasible to achieve high concentrations of certain drugs in the vitreous space and therefore, anticipate a local biological effect to address ocular pathologies.

Specific physiochemical reactions for a drug or combination of drugs must be considered when one administers medications intravitreally. Another major consideration is related to the biological effect that a drug or the combination of multiple drugs can induce in ocular tissues. Such a biological effect may be the result of tissue reactions to each individual drug or the products of their combination.

The physiochemical adverse reactions of a single, “approved” drug are usually mapped out and generally deemed safe to apply due to extensive preclinical and clinical testing that is undertaken prior to approval. Nevertheless, vigilance is still required when a single drug is being used “off-label” for a different indication. The challenge becomes even greater when two or more drugs are combined and their combination may cause new physiochemical reactions within the mixture *in vitro* or within the organ. Such adverse effects may manifest as incompatibility, which is described as preventable or reversible precipitation or insolubility and recognizable as crystals, turbidity, or haziness. Incompatibility can be easily caused by acid-base reactions. However, other scenarios are also possible, such as non-dissociated salts of organic ions, salting out, salts of inorganic divalent ions, desolvation of non-ionized organic drugs, and organic ion-inorganic ion salts.^17^

Another possible physiochemical reaction due to combinations of drugs stems from instability, which can result from hydrolysis and oxidation. The combination of chemical entities can cause degradation products and such products are often unstable chemicals that may be less active or inactive therapeutically, but more importantly, they can become toxic products which are not always visually discernable.^18^ Degradation can be caused either chemically (by factors such as concentration dependence, solution pH, photochemical, or insoluble salts) and/or physically (e.g., adsorption which is the loss of drug via adhesion to solid surfaces, complexation of ions such as calcium with tetracycline, or salting out by strong electrolytes).

As mentioned, resultant physiochemical drug interactions can become toxic and therefore, when contemplating new medications for intraocular administration, care should be given to such adverse side effects. Different cell layers of the retina, the endothelium of the cornea, and the crystalline lens are among the ocular tissues that have been reported to be subject to toxicity as a result of drug degradation or complexation products. One example of such an adverse effect was reported during cataract surgery while mixing chondroitin sulfate with hyaluronate sodium (Viscoat; Alcon Laboratories Inc., Fort Worth, TX, USA).^19^ The high concentration of phosphate in Viscoat apparently combined with calcium from the intracameral irrigating solution, resulting in clinically visible precipitation. There are also reports on clinically significant crystalline deposits on intraocular lenses with the use of Healon GV (a high concentration, high molecular-weight sodium hyaluronate).^20^ It seems that phosphate components used to buffer the viscoelastic agents precipitates with calcium from the irrigating solution. Another group reported precipitation of tissue plasminogen activator as a result of salt formation due to calcium and magnesium in the balanced salt solution (BSS) reacting with phosphate anions.^21^

The neutrality of pH for medical formulations while desirable, is a hard target to achieve for many compounds. Many formulations become unstable and suffer from lack of (partial) solubility if pushed toward a fixed pH. Ciprofloxacin is a fair example that can be stable and soluble at a pH around 4.5, but would start precipitating if pH is altered to 7.0.

Episodes of white precipitation on the corneal surface were reported among more than 600 patients with bacterial keratitis, treated with topical ciprofloxacin 0.3%.^22^ The adverse effect became more alarming when it was concluded that this precipitate was delaying re-epithelialization by as much as 55%. Ciprofloxacin was the focus of another study where precipitation was reported in the vitreous (regardless of the presence of vancomycin) at body temperature. However the amount of active drug after precipitation still remained above the MIC90 (minimum inhibitory concentration required to inhibit the growth of 90% of organisms) for most gram-negative microorganisms such that the antimicrobial effect remained intact.^23^

All of the above-mentioned examples were related to clinically visible precipitation, however, microprecipitation and/or incompatibility may also occur. A combination of vancomycin and ceftazidime has been routinely injected intravitreally for suspected bacterial endophthalmitis. There are claims of precipitation when these two antibiotics are physically mixed. Also, some investigators have reported pH-dependent precipitation of vancomycin regardless of the presence of ceftazidime in the medium.^24^

As the number of intraocular injectable candidates increases, more investigations are necessary to study such physiochemical interactions. However, designing such studies under conditions that would mimic the physiological environment of ocular tissues remains challenging. A cornerstone of maintaining such an environment is preservation of a constant internal environment in terms of temperature, fluid volume, and fluid consistency, collectively called homeostasis. One important factor in homeostasis is the maintenance of a relatively constant pH. Many buffering systems are employed in the body to support pH levels. Examples are bicarbonate in the interstitial fluid, phosphates, and proteins, which are also present intracellularly. An important source of H^+^ in the body is cellular metabolism as a result of oxidation of glucose and fatty acids. These hydrogen atoms are the most reactive cations in the body and they become crucial when one considers the functional groups of proteins, which are negatively charged and can react with them. Such reactions may cause alterations in the structural conformation of the protein and consequently its behavior. Many new drugs in development are protein-based and therefore such possible reactions with other drugs become noteworthy. Less is known about the buffering capacity of the vitreous in general, until recently.^25^ A series of experiments involving the addition of triamcinolone acetonide, sodium hydroxide, and hydrochloride acids, were designed to investigate the buffering ability of this ocular medium. In this investigation, the bovine vitreous showed superior buffering aptitude against the addition of acids and bases, when compared to a control medium such as normal saline. This superiority was as much as five times more than that of normal saline, meaning most intravitreal injectable drugs can easily be buffered by an intact vitreous.

It has been demonstrated that intraocular injections of drugs can cause toxicity to different tissues. Such toxicity is usually shown by means of postmortem histologic studies in animal models. Toxicity to retinal cell layers can be shown *in vivo* using electroretinography. Toxicity in the anterior segment such as lens opacification can easily be revealed with slit-lamp biomicroscopy. Corneal toxicity usually manifests as endothelial cell dysfunction which can be shown using a corneal stress test.^26^

Less risky methods for testing the biological effects of combination (and single) drugs in preclinical settings include bacteria, specialized cell cultures, ocular inflammatory receptors, viruses, and proliferative tissues.

Currently, therapy for bacterial endophthalmitis consists of combination therapy, a mixture of antibiotics aimed at addressing both gram-negative and gram-positive bacteria. This is the standard of care since such a sight-threatening condition allows no time to explore the nature of the causative organism. Antibiotics usually interfere with bacterial cell wall or ribosomal protein synthesis. Gram-positive bacteria have different cell walls from gram-negative microorganisms and therefore a single antibiotic may be ineffective on the wrong target. The current antibiotic treatment for acute-onset bacterial endophthalmitis includes a mixture of vancomycin for gram-positive coverage and either ceftazidime or an aminoglycoside for gram-negative coverage. Aminoglycosides do not interact with the cell wall but inhibit protein synthesis by binding to the 30S bacterial ribosomal subunit, resulting in misreading of the messenger RNA. Toxicity levels for each of these antibiotics have been determined and they are generally considered to be safe for administration through the pars plana.^27^–^37^

When Peyman and colleagues first introduced combination therapy for posterior segment disorders^15^,^27^–^29^,^38^, their goal was to directly combat the microorganisms and at the same time reduce ongoing inflammation associated with the process. Of note, bacterial infection in the ocular media is accompanied by considerable intraocular inflammation. Examples of inflammatory mediators include lipoxygenases, which produce leukotrienes, and cyclooxygenases, which produce prostaglandins and thromboxanes. Lipoxygenases are mainly inhibited by steroids, whereas cyclooxygenases are inhibited by non-steroidal anti-inflammatory compounds (NSAIDs).

Combinations of steroids and amphotericin B have been investigated for fungal infections which are also typically accompanied by marked inflammation.^39^ Results have shown that significant reduction in intraocular inflammation can be achieved with combination therapy in comparison to single agents or no treatment.^40^,^41^

In current practice, corticosteroids are important candidates for intravitreal applications. Non-toxic doses of dexamethasone for ocular tissues are close to 400 μg^40^. Currently, dexamethasone is routinely administered at a dose of 400 μg, which is markedly lower than the subconjunctival dose at 2 to 5 mg, or systemic dose at 75 mg. However, dexamethasone phosphate is a water soluble salt that can be eliminated from the vitreous cavity within a few days. It thus has a half-life of approximately 3 hours (the biological half-life is more than 40 hours). Recent intravitreal and subconjunctival pharmacokinetic studies mapped this fast elimination in a rabbit study *in vivo*.^42^ Triamcinolone acetonide, which has a half-life of 18.6 days, is now the candidate of choice to sustain longer residence in ocular tissue. The usual dose for intravitreal administration is 2 to 4 mg, although higher doses have also been explored with success.^41^,^43^–^48^ Due to the long acting nature of triamcinolone, it is an acceptable choice for treatment of conditions such as cystoid macular edema (CME) and many other persistent posterior segment inflammatory conditions. One major reservation for intraocular application of steroids relates to their side effects including cataract formation and elevation of intraocular pressure.^49^

A triple combination of clindamycin, aminoglycosides, and dexamethasone in the infusion fluid has been investigated by our group since 1990 for prophylaxis of bacterial infection in vitrectomy procedures.^50^

## COMBINATIONS OF DRUG AND DEVICE

A combination therapy, per regulatory guidelines, can include a device and a drug. Steroids have been successfully formulated and incorporated into implantable devices. Retisert is an example of a drug and device combination, engineered as a non-biodegradable intravitreal implant for slow but steady release of fluocinolone acentonide for up to 3 years. The device is sutured into the vitreous cavity, away from the optical axis and acts as a steady drug delivery system.^49^ The high incidence of cataract formation and intraocular pressure elevation in patients receiving this implant were among the reasons for its lack of endorsement by leading ophthalmologists. In 2010, the FDA approved Ozurdex, which is a dexamethasone intravitreal implant for treatment of adults with macular edema following branch retinal vein occlusion (BRVO) or central retinal vein occlusion (CRVO). Ophthalmologists use this implant for uveitis and other non-infectious inflammations of the posterior segment. This is an example of a specialized drug depot combined with a device that injects a biodegradable amount of a special dexamethasone formulation for slow and steady release.^51^ Its approval was based on data from a 26-week multicenter double-blind randomized clinical study in which 77 patients received Ozurdex 0.7 mg while 76 patients received sham injections. Although such a novel drug delivery platform is exciting from a scientific point of view, critics may question the real world utility of a steroid depot formulation as the ideal treatment for such indications. Over the past several years, clinicians have used intraocular injections of triamcinolone acetonide (Kenalog) off-label for the treatment of macular edema due to BRVO and CRVO. Ozurdex is designed for a sustained release of dexamethasone but at a much higher price compared to triamcinolone acetonide.

Another interesting vehicle for delivery of steroids is iontophoresis technology. Iontophoresis utilizes an electric current to drive charged ions (drugs) across a barrier such as the sclera or the cornea. Eyegate Pharmaceuticals has recently completed a phase II study on management of anterior uveitis using an iontophoretic drug delivery system for ocular application.^52^ The safety, tolerability, and efficacy of four iontophoretic doses of dexamethasone phosphate ophthalmic solution were investigated in patients with non-infectious anterior segment uveitis. The technology, however, has certain limitations; for example, the number of charges on a given drug ion partially determines its mobility. Also, the drug’s molecular size will affect its transport kinetics and other competing ions in the formulation or tissue could adversely affect the delivery profile.

Other delivery devices are in development to address on-target transport challenges that new drugs and therapies face to reach the posterior segment. A novel injection device for intravitreal and subconjunctival drug delivery has been developed by Peyman and co-workers^53^. The device utilizes a micro-needle to “hook” onto the ocular surface; the injection of medication into the vitreous cavity is achieved by propelling the formulation using the jet force produced by device internals. The device has a number of advantages: the actual injection time is a fraction of a second while the whole procedure takes a few seconds; the pars plana is predetermined by placing the tip of the probe adjacent to the limbus; and the procedure can potentially be executed by a general ophthalmologist. Furthermore, there is no needle penetration into the vitreous cavity, which eliminates the danger of abrupt eye movement during the injection phase. This should also theoretically lower the chances of microflora transfer from the ocular surface into the eye, minimizing the risk of endophthalmitis. Additionally, preclinical studies suggest that there is no drug regurgitation, which frequently occurs after intravitreal and subconjunctival injections (unpublished data).

Viral infections of the posterior segment have also been targeted with combination therapies. Viral infections develop in different stages and most antiviral drugs interfere with the replication stage. They do so by integrating defective nucleoside analogues into the viral genome and thus, targeting specific viral enzymes or proteins. Fortunately the incidence of cytomegalovirus retinopathy is decreasing thanks in part to the employment of advanced therapies for HIV infected individuals. Nevertheless, antiviral therapies for this condition are still being used, and a drug and device combination (Vitrasert) for intravitreal implantation is available for slow release of ganciclovir up to 8 months. This implant was developed before Retisert and its implantation procedure resembles that of Retisert. Antiviral drugs were injected intravitreally before the introduction of implants. Different doses were used to administer each compound, for example, ganciclovir at 2000 μg, foscarnet at 1000 to 2000 μg, and acyclovir at 240 μg. Other antiviral drugs such as trifluorothymidine and vidarabine have also been injected intravitreally. A combination of ganciclovir and foscarnet (or other antiviral drugs) can also be used for mixed therapy.

## IMPLICATIONS OF COMBINATION THERAPY

Several ocular pathologies could benefit from combination therapies. Diseases such as proliferative vitreoretinopathy (PVR), vascular proliferation in age-related macular degeneration (AMD), diabetic retinopathy, and malignant tumors, exhibit distinctly different stages which can be potential targets for different anti-proliferative, anti-inflammatory, and matrix metalloproteinase inhibiting agents. Current management of vascular proliferation has been dominated by blocking the activity of existing vascular endothelial growth factor (VEGF), while other small and large molecular candidates are being investigated to block the production of VEGF. Targeting VEGF in AMD has probably provided the biggest advance for this ever-growing condition. Currently, the two prominent treatment options for VEGF blockage include intravitreal administration of ranibizumab (Lucentis) or the off-label use of bevacizumab (Avastin). However, other VEGF blocking treatments are in development and probably the closest compound to approval is aflibercept (VEGF Trap), developed by Regeneron Pharmaceuticals. Aflibercept is a fusion protein specifically designed to bind all forms of vascular endothelial growth factor-A (called VEGF-A).

A number of candidates for co-administration with VEGF blocking agents are being investigated clinically and results thus far are promising. These include not only intravitreal combinations of drug candidates but also intravitreal and topical combinations aimed at increasing efficacy and/or decreasing injection frequency and cost, thus ultimately improving compliance. In one clinical study, the combination of topical bromfenac 0.09% (Xibrom, Ista Pharmaceuticals Inc., Irvine, CA, USA), which is an NSAID, and the intravitreal injection of ranibizumab resulted in a threefold decrease in the frequency of injections.^54^ Another study could not replicate these results but the authors pointed out that their follow-up period was only 2 months, as opposed to 6 months in the original study.^55^ Another NSAID, which was recently combined in a clinical setting with intravitreal injections of anti-VEGF compounds, is nepafenac (Nevanac) for treatment of recalcitrant exudative macular degeneration. Based on a 3 month study, the authors reported no significant change in visual acuity or quantitative optical coherence tomography (OCT) measurements but noted a trend toward improved anatomy and qualitative OCT findings when topical nepafenac was added to monthly anti-VEGF injections in patients with persistent intraretinal cysts, subretinal fluid, and pigment epithelial detachment.^56^

Other therapies that can be combined with anti-VEGF therapy include photodynamic therapy (PDT), transpupillary thermotherapy (TTT), and intravitreal steroid injections. There have been a number of uncontrolled clinical studies where combinations of VEGF therapy with steroids and PDT (as a combo or triple treatment) were investigated, with favorable results. Triple therapy for posterior segment diseases such as wet type AMD has been investigated by a number of clinicians.^57^–^59^ It involves a treatment protocol with intravitreal injections of a steroid such as dexamethasone or triamcinolone, and an anti-VEGF compound such as pegaptanib (Macugen), bevacizumab, or ranibizumab, followed by short-duration photodynamic therapy that can stabilize vision and reduce the burden of frequent injections. However, it is still unclear which VEGF forms should be blocked for each disease and to what extent should such blockade be pursued, since VEGF also has physiologic roles for many processes in the body, such as wound healing, survival, and regeneration of healthy cells.

## CONCLUSION

Combination therapies are increasingly expanding due to the growth of knowledge about the core pathologic mechanisms of diseases. Ophthalmology has been an area of successful implementation of a number of fixed and unfixed drug combinations, along with drug/device combinations. Investigators have combined different drugs with devices to address challenging needs of chronic conditions and drug delivery problems. Such combination approaches should ideally achieve clear advantages in terms of therapeutic efficacy, lowered toxicity, and increased compliance. There are also risks that need to be mitigated, if possible. Combinations of pharmaceutical compounds may precipitate adverse physical and chemical interactions, both *in vitro* during mixing and *in vivo* upon delivery. Although research on adverse effects of drug/drug and drug/device interactions is regulated for commercial combinations, there is little control over off-label and unfixed combinations devised and utilized by clinicians. More research is warranted on new combination therapies to increase efficacy and safety.
